# Ten modifiers of *BRCA1* penetrance validated in a Norwegian series

**DOI:** 10.1186/s13053-015-0035-0

**Published:** 2015-05-30

**Authors:** Cecilie Heramb, Per Olaf Ekstrøm, Kukatharmini Tharmaratnam, Eivind Hovig, Pål Møller, Lovise Mæhle

**Affiliations:** 1 Research Group on Inherited Cancer, Department of Medical Genetics, Oslo University Hospital, The Norwegian Radium Hospital, Oslo, Norway; 2 Department of Tumor Biology, Institute for Cancer Research The Norwegian Radium Hospital, Oslo, Norway; 3 Department of Mathematics, University of Oslo, Blindern, Oslo Norway

**Keywords:** BRCA1, Modifiers, Genetic drift

## Abstract

**Background:**

Common genetic variants have been shown to modify *BRCA1* penetrance. The aim of this study was to validate these reports in a special cohort of Norwegian *BRCA1* mutation carriers that were selected for their extreme age of onset of disease.

**Methods:**

The ten variants rs13387042, rs3803662, rs8170, rs9397435, rs700518, rs10046, rs3834129, rs1045485, rs2363956 and rs16942 were selected to be tested on samples from our biobank. We selected female *BRCA1* mutation carriers having had a diagnosis of breast or ovarian cancer below 40 years of age (young cancer group, *N* = 40), and mutation carriers having had neither breast nor ovarian cancer above 60 years of age (i.e., old no cancer group, *N* = 38). Relative risks and odd ratios of belonging to the young cancer versus old no cancer groups were calculated as a function of having or not having the SNPs in question.

**Results:**

Five of the ten variants were found to be significantly associated with early onset cancer. Some of the variation between our results and those previously reported may be ascribed to stochastic effects in our limited number of patient studies, and/or genetic drift in linkage disequilibrium in the genetically isolated Norwegian population. This is in accordance with the understanding that the SNPs are markers in linkage disequilibrium with their respective disease-causing genetic variants, and that this may vary between different populations.

**Conclusions:**

The results confirmed associations previously reported, with the notion that the degree of association may differ between other populations, which must be considered when discussing the clinical use of the associations described.

**Electronic supplementary material:**

The online version of this article (doi:10.1186/s13053-015-0035-0) contains supplementary material, which is available to authorized users.

## Introduction

Mutations in the *BRCA1* gene constitute a high life-time risk of breast and ovarian cancer. Risk reducing salpingo-oophorectomy over the age of 35 years is advocated to reduce the risk of cancer and early death [1]. Breast cancer may be prevented by prophylactic mastectomy and patient prognosis improved when breast cancer is detected early with mammography and MRI [2]. Because *BRCA1*-associated breast cancer has an early onset, prophylactic mastectomy must be undertaken at younger age to provide a maximum protective effect [3].

*BRCA1*-associated cancer is age-dependent, and whether or not this is stochastic or influenced by other factors (modifiers of penetrance) is a question that has not been fully explored: Both stochastic elements and modifying factors may be instrumental in diseases causation. Modifying factors may be genetic, environmental, or both. This study was designed to validate previous reports of normal genetic variants that contribute to modifying *BRCA1* penetrance.

A number of normal single nucleotide polymorphisms (SNPs) associated with breast cancer in the general population have been demonstrated to modify the penetrance of *BRCA1*[4]–[13]. We decided not to participate in the initial studies of these modifiers of breast cancer penetrance, and we now have one of the few sufficiently large series of well-described *BRCA1* mutation carriers to validate the findings reported by others.

The aim of this study was to determine whether SNPs reported to be associated with cancer risk in *BRCA1* mutation carriers in other populations had the same association in the Norwegian population.

## Materials and methods

### Selection of patients

All study subjects were demonstrated *BRCA1* mutation carriers at the outpatient Cancer Genetics Clinic, Oslo University Hospital and the respective mutations were as previously reported [14]. Two groups were selected for analysis: Mutation carriers having had breast and/or ovarian cancer under the age of 40 years, hereafter described as the young cancer group, and mutation carriers who were completely disease free until their 60^th^ birthday, or older (the old no cancer group). If there were to be significant associations between age at onset of cancer and the SNPs considered in our material, they should be identified by comparing the extremes by this approach. The study was approved by both the Ethical review board (ref S02030) and the Norwegian Data Inspectorate (ref 2001/2988-2). All genetic counseling and testing was performed according to Norwegian law, and all patients gave written informed consent. The present report is one in a series to meet a request from the Norwegian Parliament to report the results of our studies into inherited breast and or ovarian cancer risk. We did not discriminate between breast or ovarian cancer, as we have previously shown that there is no sib pair concordance for breast and ovarian cancer in *BRCA1* mutation carriers in our population [15]. Patients having had prophylactic mastectomies under the age of 60 years were excluded from the study, patients having had prophylactic salpingo-oophorectomy, but not mastectomy, were included in the old group. Power calculations indicated that, if reasonable prevalence of the variant alleles for each of the modifiers tested, we would reach significance if the OR > 2 or <0.5, and with 50 participants in both the young cancer and old no cancer groups. Preliminary analyses on the number of women having consented to participate, indicated that we would reach significance by selecting affected women aged less than 40 years and women unaffected at over 60 years as mentioned previously.

### Test panel/selection of SNPs

Not knowing the prevalence of the genetic variants in question in Norway, nor their association with disease, we selected the ten genetic variants reported to have the highest association with early/late onset of breast cancer among *BRCA1* mutation carriers in 2011 when the study was designed. The test panel consisted of nine single nucleotide polymorphisms (SNPs) and one deletion shown to be associated with cancer risk in *BRCA1* mutation carriers as shown in Table 1. For simplicity, we will in this report refer to the deletion as a SNP. Seven of the SNPs were reported to increase cancer risk [4]–[10] and three were reported to decrease risk for breast cancer in *BRCA1*-mutation carriers [11]–[13].

**Table 1:** Distribution of genotypes in the ten SNPs determined in *BRCA1* mutation carriers with breast or ovarian cancer before 40 years of age (young cancer) and in carriers not having had breast or ovarian cancer before 60 years of age (old no cancer), and with calculated RR and OR, and HR/OR from previous reports [according to references given in left column]

SNP	Genotype	Young cancer (number of cases)	Old no cancer (number of cases)	Reported risk	ObservedRR (95 % CI)	ObservedOR (95 % CI)	Fishers’ exact p – one sided
rs13387042 2q35 [4]	GG	13	18		1	-	
	AA	9	9	HR 1.05	1.19 (0.64–2.22)	1.38 (0.43–4.45)	0.40
	GA	17	11	HR 1.14	1.45 (0.87–2.41)	2.14 (0.76–6.06)	0.12
	AA or GA	26	20		1.35 (0.83–2.19)	1.80 (0.72–4.52)	0.15
rs3803662 16q12 TOX3, LOC643714 [5]	CC	18	26		1	-	
	TT	1	2	HR 1.24	0.81 (0.16–4.20)	0.72 (0.06–8.58)	0.65
	CT	20	10	HR 1.11	1.63 (1.05–2.52)	2.89 (1.10–7.61)	0.03*
	TT or CT	21	12		1.56 (1.00–2.41)	2.53 (1.00–6.40)	0.04*
rs8170 19p13 [6]	GG	29	27		1	-	
	AA	0	1	HR 1.35	-	-	-
	GA	11	10	HR 1.22	1.01 (0.63–1.63)	1.02 (0.38–2.80)	0.58
	AA or GA	11	11		0.97 (0.59–1.57)	0.93 (0.35–2.50)	0.65
rs9397435 6q25 ESR1 [7]	AA	32	34		1	-	
	GG	1	0	HR 1.37	2.06 (1.61–2.64)	-	-
	AG	7	4	HR 1.31	1.31 (0.79–2.19)	1.86 (0.50–6.96)	0.27
	GG or AG	8	4		1.38 (0.86–2.20)	2.13 (0.58–7.75)	0.20
rs700518 CYP19 [8]	AA	12	14		1	-	
	GG	16	6	OR 2.81	1.58 (0.97–2.57)	3.11 (0.92–10.48)	0.06
	AG	11	17	OR 1.41	0.85 (0.46–1.58)	0.75 (0.26–2.23)	0.41
	GG or AG	27	23		1.17 (0.72–1.91)	1.37 (0.53–3.54)	0.34
rs10046 CYP19 [9]	CC	11	15		1	-	
	TT	17	7	OR 1.37	1.67 (1.00–2.81)	3.31 (1.02–10.72)	0.04*
	TC	12	15	OR 1.26	1.05 (0.57–1.94)	1.09 (0.37–3.23)	0.55
	TT or TC	29	22	0R 1.29	1.34 (0.81–2.23)	1.80 (0.69–4.67)	0.17
rs3834129 CASP 8 [10]	nor/nor	8	9		1	-	
	del/del	12	6	HR 1.60	1.42 (0.78–2.58)	2.25 (0.57–8.82)	0.20
	nor/del	19	23	HR 1.83	0.96 (0.53–1.76)	0.93 (0.30–2.88)	0.56
	del/del or nor/del	31	29		1.10 (0.63–1.92)	1.20 (0.41–3.54)	0.48
rs1045485 CASP 8 [11]	GG	33	25		1	-	
	CC	1	6	HR 0.86	0.25 (0.04–1.56)	0.13 (0.01–1.12)	0.04*
	GC	5	5	HR 0.83	0.88 (0.45–1.70)	0.76 (0.20─2.91)	0.47
	CC or GC	6	11		0.62 (0.31─1.23)	0.41 (0.13─1.27)	0.10
rs2363956 9p13 ABHD8, ANKLE1, C19orf62 [12]	AA	15	5		1	-	
	CC	7	13	HR 0.7	0.47 (0.24–0.89)	0.18 (0.05–0.70)	0.01*
	AC	18	19	HR 0.89	0.65 (0.43–0.98)	0.32 (0.10–1.05)	0.05*
	CC or AC	25	32		0.58 (0.40–0.86)	0.26 (0.08–0.81)	0.02*
rs16942 BRCA1 [13]	TT	20	23		1	-	
	CC	5	9	HR 0.85	0.77 (0.35–1.66)	0.64 (0.18–2.22)	0.35
	TC	15	6		1.54 (1.01–2.34)	2.88 (0.94–8.82)	0.05*
	CC or TC	20	15		1.23 (0.80–1.89)	1.53 (0.62–3.77)	0.24

*: *p* < = 0.05

Initially, we demonstrated the SNPs in the test panel to be polymorphic in our population of healthy Norwegian blood donors (*N* = 3000), and the rare SNP alleles had a frequency > 5 % (data not shown). The disease-associated alleles were defined as the minor or risk allele (from which positive or negative associations with disease were calculated), regardless of whether or not this was the least common allele in our population.

### Genotyping

#### Samples

Blood samples were obtained after informed consent and stored at −20 °C (or −70 °C). DNA was extracted from 200 μl of whole blood by using a Qiagen BioRobot M48 Robotic Workstation, following the protocol of the MagAttract DNA Blood Mini M48 kit (Qiagen, Hilden, Germany).

#### Fragment design

Default Primer3 (http://bioinfo.ut.ee/primer3/, last accession date 03062014) parameters were applied when designing primers used to amplify fragments around each DNA variant, identified by the NCBI SNP reference numbers (rs) [16]. A 42-bp artificial high melting domain, labeled with 6-FAM, was incorporated at one end of the amplified target using a set of three primers in the PCR setup [17].

#### PCR

The PCR reaction mixtures were as described by the manufacturer (Life technologies Carlsbad CA) without modification. Annealing temperatures are given in Additional file 1.

#### Electrophoresis

We used cycling temperature capillary electrophoresis (CTCE) to detect allelic variants as described previously [16], [17].

### Statistics

We confirmed that the prevalence of the SNPs in the young cancer and old no cancer groups assessed together were all in Hardy–Weinberg equilibrium.

Since this was a one-sided study, we used Fishers’ exact to identify any significant association.

## Results

The selection criteria applied to our data set revealed 40 patients in the young cancer group and 38 participants in the old no cancer group, which was considered sufficient to reveal any difference in the frequency of SNPs between the two groups. Forty-seven (60 %) patients had eight different founder mutations previously reported [18], of whom 25 belonged to the young onset cancer group. Thirty-one (40 %) had altogether 19 different mutations, of which 15 had young onset cancer. The observed results and the calculated RRs, ORs and significance levels are shown in Table 1, together with the previously reported HRs and ORs [4]–[13]. All ten SNPs tested showed point estimates of being positively or negatively associated with having early onset breast cancer similar to previous reports. Because the references had used different ways of ascertaining patients, including different methods by which to calculate HRs and ORs, we had no exact notion of what RRs and ORs would be calculated for our study, and could not compute theoretical significance levels against those expected.

We found that rs3803662 was significantly associated with early onset breast cancer (*p* = 0.026 for heterozygous cases and *p* = 0.040 for homo–or heterozygous cases). The SNP rs10046 was positively associated with early onset disease in the homozygous state (*p* = 0.040), and rs104585 was negatively associated with early onset breast cancer (*p* = 0.039 for homozygous). The SNP rs2363956 was negatively associated with early cancer (*p* = 0.012, *p* = 0.049 and *p* = 0.015 for homo-, hetero- or homo- or heterozygous, respectively). Finally, rs16942 was significantly associated with early onset breast cancer (*p* = 0.05 for heterozygous). The distributions for homozygous versus heterozygous for rs16942 were conflicting and remain to be precisely defined. The rs16942 SNP is within linkage distance from the BRCA1 gene, and haplotyping of the patients/families in question may be necessary to consider this further.

## Discussion

In principle, we have confirmed the reported association between the presence of variant SNPs and early onset of breast cancer in *BRCA1* mutation carriers. Five of the SNPs tested revealed significant associations with early ages of onset cancer, whereas five did not. The lack of association may be due to different associations in the Norwegian population compared to other populations, which may be a result of genetic drift [18]. Also, stochastic variation in our restricted number of patients may have obscured the associations examined.

Sixty-three percent of the mutation carriers included had one of the frequent Norwegian founder mutations, in which we have determined the penetrance to be similar in retrospective series of extended pedigrees, which we later confirmed through prospective studies of new cases in the same families [18], [19]. There were no associations with the presence of founder mutations or less frequent mutations with early onset cancer. For this reason, we do not have the confounder of putative different penetrance of the causative mutations as discussed in other reports [4]–[13]. This may be a third possible cause for a stronger association in the Norwegian population. Yet another possible cause for the stronger associations in our population is that we scored both breast and ovarian cancer as affected phenotypes, while most reports considered only breast cancer.

Also, most previous reports calculated HR from continuous distribution by other methods. The described differences between the young cancer cases and the old no cancer group might have been expected to show stronger associations than those previously reported [4]–[13], due to the methods applied, and not because of differences in the populations studied.

We find that the limited number of cases in our study, and some discrepancies between the previously reported distribution between homozygous and heterozygous carriers in comparison to our findings, is likely to result in insufficient power to evaluate the underlying mechanisms of the associations observed. Our results may, however, be considered to contribute towards a future combined effort to precisely define the contribution of risk provided by these polymorphisms. We would like to add, however, that some of the discrepancies found may not be methodological artifacts, but rather related to differences in linkage disequilibrium between the SNPs studied and disease in different populations. If this is verified, the search for an actual risk value for the association between breast cancer and the presence or absence of a given SNP that is population specific may be a useful approach in risk stratification. Some of the slight variations in associations reported in the different populations may have been caused by such mechanisms, commonly referred to as genetic drift.

In conclusion, our validation gave similar, but not identical results compared to those published by others.

Also, it is not currently established whether or not the association to these SNPs are of clinical interest. We have previously shown that BRCA1 carriers in our population have on average 25 % risk of developing breast cancer at 40 years of age [19]. The associations reported here may give a ten percent higher or lower cancer risk estimate at the time. Calculating the combined modifying effects will apply to a very few cases, and the majority will be close to 25 %. The clinical utility of the findings is a question we leave open for discussion. Through this report, we make the findings available for *BRCA1* mutation carriers in our population and for international meta-analyses.

## Additional file

Below is the link to the electronic supplementary material.

## Electronic supplementary material

Additional file 1: Annealing temperatures PCR. (DOC 32 kb) (DOC 32 KB)
