# Supplementary material for: Ten modifiers of BRCA1 penetrance validated in a Norwegian series
Source: Hered Cancer Clin Pract. 2015 May 30;13:14. doi: 10.1186/s13053-015-0035-0 (PMC4456774; doi:10.1186/s13053-015-0035-0)
Supplement: Supplementary file 1 — Additional file 1: Annealing temperatures PCR. (DOC 32 kb) (DOC 32 KB) [file 13053_2015_35_MOESM1_ESM.doc]

**Supplementary material**

**Annealing temperatures PCR**

| **Variant (location,** gene**)** | **Annealing temperature °***C* |
| --- | --- |
| **rs13387042 (2q35)** | 63 |
| **rs3803662 (16q12** *TOX3, LOC643714)* | 62.5 |
| **rs8170 (19p13)** | 63 |
| **rs9397435 (6q25** *ESR1)* | 63 |
| **rs700518 (***CYP19)* | 63 |
| **rs10046 (***CYP19)* | 68 |
| **rs3834129 (***CASP8)* | 67 |
| **rs1045485 (***CASP8)* | 63 |
| **rs2363956 (19p13** *ABHD8, ANKLE1, C19orf62)* | 62.5 |
| **rs16942 (***BRCA1)* | 65 |
